# Improving COVID-19 vaccine uptake: a message co-design process for a national mHealth intervention in Colombia

**DOI:** 10.1080/16549716.2023.2242670

**Published:** 2023-08-29

**Authors:** Nathaly Aya Pastrana, Sandra Agudelo-Londoño, Oscar Franco-Suarez, Jessica Otero Machuca, Deivis Nicolás Guzman-Tordecilla, María Camila López Sánchez, Mariana Rodriguez-Patarroyo, Cristhian Alejandro Rivera-Sánchez, Daniella Castro-Barbudo, Antonio J. Trujillo, Vidhi Maniar, Andres I. Vecino-Ortiz

**Affiliations:** a IMEK Centro de Investigación en Mercadeo & Desarrollo, Santiago de Cali, Colombia; b Instituto de Salud Pública, Pontificia Universidad Javeriana, Bogotá, Colombia; c Health Behavior, Gillings School of Global Public Health, University of North Carolina at Chapel Hill, Chapel Hill, NC, USA; d Department of International Health, Johns Hopkins Bloomberg School of Public Health, Baltimore, MD, USA

**Keywords:** Co-design, intervention development, COVID-19 vaccination, behavior change, message design

## Abstract

**Background:**

COVID-19 vaccination is a global priority. Latin American countries have some of the highest COVID-19 death rates worldwide with vaccination hampered by a variety of reasons, including mis- and disinformation, vaccine hesitancy, and vaccine supply constraints. Addressing vaccine hesitancy through effective messages has been found to help increase vaccine uptake. Participatory processes could be used to co-design health messages for this purpose.

**Objective:**

This article describes the methodology used to co-design evidence-based audio messages to be deployed in a cohort of individuals through an interactive voice response (IVR) mobile phone survey intervention, aimed towards increasing vaccination uptake in an adult population in Colombia.

**Methods:**

Participants of the COVID-19 vaccination message co-design process included a sample of the general population of the country, representatives of the funder organisation, and research team members. The co-design process consisted of four phases: (1) formative quantitative and qualitative research, (2) message drafting based on the results of the formative research, (3) message content evaluation, and (4) evaluation of the voices to deliver the audio messages; and was informed by reflexive meetings.

**Results:**

Three categories of evidence-based audio messages were co-designed, each corresponding to an arm of the mHealth intervention: (1) factual messages, (2) narrative messages, and (3) mixed messages. An additional fourth arm with no message was proposed for control. The iterative co-design process ended with a total of 14 audio messages recorded to be deployed via the intervention.

**Conclusions:**

Co-developing health messages in response to health emergencies is possible. Adopting more context-relevant, participatory, people-centred, and reflexive multidisciplinary approaches could help develop solutions that are more responsive to the needs of populations and public health priorities. Investing resources in message co-design is deemed to have a greater potential for influencing behaviours and improving health outcomes.

## Introduction

Improving COVID-19 vaccine uptake is a priority worldwide. Globally, more than 528 million cases of COVID-19 have been reported since the beginning of the pandemic up to August 2022 [1] At a regional level, COVID-19 death rates in Latin America are among the highest globally [2] As of December 2022, over 1.34 million COVID-19 deaths had been reported in South America [3], with over 141,881 deaths in Colombia [4]. Vaccination against COVID-19 can prevent severe illness and reduce hospitalisations and deaths [5]. COVID-19 vaccination rates vary across regions and countries [6], for example, in Colombia, by 9 August 2022, a total of 87,566,541 free COVID-19 vaccine doses had been administered [7] representing about 82% of the population with at least one dose [8]. Nevertheless, COVID-19 vaccine uptake has been hampered by mis- and disinformation, vaccine hesitancy, and vaccine supply constraints [9–11]. These factors have been influenced by the historic mishandling of public health initiatives and structural constraints on the public health infrastructure [12].

Misperceptions and poor communication around vaccination can contribute to vaccine hesitancy [13]; however, developing strategies to address these factors can help increase vaccine uptake [13]. As vaccination coverage increases, individuals with higher hesitancy become a priority, and behaviour change strategies become more relevant. Such strategies often use communication and social marketing principles, concepts, and frameworks to motivate voluntary behaviour change [14]. For example, the SAGE Working Group on Vaccine Hesitancy has recommended social marketing to address vaccine hesitancy, emphasising the importance of the concept of ‘value’ to motivate people to get vaccinated [14]. Communication and social marketing behaviour change interventions are more effective when they are guided by theory [13,15], and when they are adapted to current local contexts. Understanding the historical and relational processes affecting the social order of a specific context, and the effect of people’s characteristics and experiences, which are, in part, determined by their social identity factors (e.g. gender, beliefs, geographic location) [14,16] and emotional determinants [2], on the uptake of behaviour is considered key to designing effective behaviour change interventions. This is particularly relevant to the context of Colombia, where its 51 million inhabitants are dispersed in diverse geographical areas (e.g. regions, departments) with varying social, cultural, and economic characteristics, previous vaccination experiences, and accessibility to government services [17,18]. Consequently, strategies to address vaccine hesitancy and incentivise vaccine uptake in Colombia should consider this diversity.

Messages to incentivise the voluntary uptake of COVID-19 vaccines should provide more than just information. Tironi et al. [19] suggested that as the rates of vaccination increase, results of vaccine uptake should also be made observable by showing people that their peers have been vaccinated or by making visible the aggregate rates of vaccination uptake, in addition to communicating that the COVID-19 vaccines are safe and effective. However, while communicating, evidence-based health information is necessary for an effective public health response [20,21], since people do not always process health-related information rationally [22,23]. Using an empathic communication style is crucial to address misinformation, obtaining the attention of the audience of focus, and addressing their concerns [9]. The literature shows that message tactics (e.g. framing) affect intentions to adhere to a recommended health behaviour [14,22,24]. Using emotional appeals that emphasise the social and physical consequences of a health issue can help influence behaviours [24]. However, these emotional appeals should be used with care and respect, given the impact of COVID-19 on the physical, emotional, and mental health of people [25,26], and the particular social and economic effects of the pandemic in the world [27].

Messages should be relevant to people’s experiences and provide practical steps that are easy to understand for broader audiences [20]. Effective communication is characterised by being timely, accurate, clear, concise, credible, and relevant [20,21,28]. It should also be sufficient, consistent, and understandable [20]. When designing health messages, it is necessary to be cognisant of the content, how it is presented [22], the message source, the mode of delivery, and their potential impact on people’s trust [13,22]. However, the content of the messages and the communication channels used to disseminate them are not the only factors influencing the acceptance of a message [23]. A person’s worldviews, sense of autonomy and liberty to make choices, conceptions of meaning, values, and how they envision responsibility are also important to consider when devising health messages aiming to motivate voluntary behaviour change [23]. It is for this reason that participatory processes are key to improving the content of health messages [29,30].

The literature shows that mobile health (mHealth) interventions co-designed with the participation of different stakeholders, instead of solely scientists, may be more effective, especially when involving the audiences of focus [31]. Defined as ‘the emerging mobile communication and network technologies for healthcare systems’ [32], mHealth interventions are cost-effective to use in emergency contexts and with remote populations [33]. An mHealth technology that has led to health improvements across countries is interactive voice response (IVR), an automated system using pre-recorded voice messages where participants can answer calls by pressing the keys of their telephone without synchronic interaction with an interviewer [34]. While IVR has been successfully used in low- and middle-income countries (LMICs) [35,36], there is limited evidence about how it has been used for participatory processes. Methods often used to facilitate user participation encompass surveys, interviews, advisory team discussions, storyboarding, piloting to understand user acceptability, and workshops [31]. The evidence indicates that similar co-design methods have been effective in developing health interventions in Latin American settings [29,37].

Evidence supporting the processes used to develop COVID-19 vaccination messages is sparse. Overall, much of the research on the COVID-19 pandemic has focused on other matters, such as responses to the outbreak [21,38,39], epidemiological surveillance [40], vaccine development processes, and barriers and drivers of vaccination [19,41]. Additionally, there is a dearth of evidence of the processes that have been used to develop messages to incentivise vaccine uptake in general, and COVID-19 vaccine uptake in particular, despite the existence of evidence underlining the ability of participatory methods to increase vaccine uptake [42], and the positive results that co-design processes have had in mHealth interventions implemented in LMICs [37]. Co-design processes might not be carried out due to time or cost limitations. This article aims to address these gaps by describing the processes used to co-design messages to promote the voluntary uptake of COVID-19 vaccines by inhabitants of Colombia. We describe the methodology by which we developed evidence-based audio messages that are to be deployed in a cohort of individuals through IVR mobile phone surveys, aiming at increasing vaccination uptake in an adult population.

## Methods

This work is part of the project entitled ‘Digital Applications to Monitor Novel Coronavirus Disease and Response in Colombia – syndromic and vaccination surveillance (DIAMOND-R)’. The DIAMOND-R project, implemented between 2021 and 2023, used IVR calls for [1] COVID-19 surveillance and [2] to promote vaccination among adult people living in Colombia. The project used both quantitative and qualitative methods to understand determinants of COVID-19 vaccine hesitancy including knowledge, perceptions, and vaccination experiences. Data collected informed the processes of co-design and evaluation of messages to incentivise vaccination uptake as described in this article. The messages developed will be nationally deployed via an mHealth intervention through IVR.

### Participants

The message co-design process included input from the general population representing the country’s regions; experts in behavioural economics from the Inter-American Development Bank (IADB), the institution that funded the DIAMOND-R project; non-local research team members, based in the United States; and local research team members, based in Colombia. It is noted that the non-local and local research team members included individuals originally from various regions of Colombia, with some of them living in cities different from the capital city of Bogota. The general population participated in formative research by providing recommendations, and in the evaluation of the voices. The behavioural economists and the non-local research team members participated in the evaluation of the content of the messages. Finally, the local research team members participated in all phases of the co-design process.

#### DIAMOND-R’s approach to co-design

**Figure 1.**
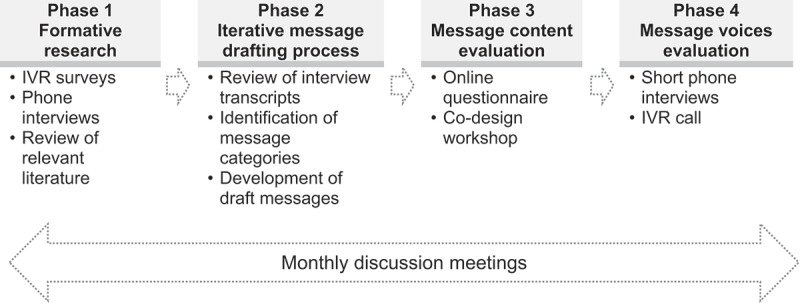
Message development process.

The process of co-designing the COVID-19 vaccination messages consisted of four phases (see Figure 1). Subsequently, the four phases of the co-design process are described.

### Phase 1: formative research

The message co-design process was informed by results from mobile phone surveys, mobile phone interviews, and a review of relevant literature. For the mobile phone surveys, the team generated a list of random Colombian mobile phone numbers and used it to call potential participants through IVR technology [43]. The surveys aimed to determine the feasibility of using mobile phone technologies to strengthen epidemiological surveillance and to improve COVID-19 vaccination among a sample of the adult population living in Colombia. The latter partially informed the development of the messages. The vaccination questions inquired about the vaccination status of participants, their willingness to get vaccinated, reasons for not being vaccinated, the number of vaccine doses received, the brands of the vaccines received, and the use of the vaccination certificate. At the end of the survey, we asked participants if they would be willing to participate in other research activities of the project. Those who were interested were invited to participate in the qualitative study.

The mobile phone interviews were carried out with a sample of participants from the mobile phone surveys, who agreed to be contacted again by the research team. This study aimed at understanding the perceptions, knowledge, and experiences around COVID-19 vaccination. The interview guide was, in part, guided by the capability, opportunity, and motivation-behaviour (COM-B) model for immunisation programmes [44,45] that was developed based on the Behaviour Change Wheel [46,47]. The COM-B model includes questions related to determinants of vaccination behaviour and groups them into three categories: capability (individual), opportunity (contextual), and motivation (individual). The interview guide was also guided by the social determinants of health [48,49] and the social marketing concept of value creation [50,51]. To design the first draft of the messages, a codebook was developed. The codes included demographic variables; vaccination status; vaccines applied; intention to get vaccinated; vaccination status of people close to the participants; positive, neutral, and negative perceptions about COVID-19 vaccines and vaccination; and suggestions made by the participants for the message design. More information on this formative research process will be provided in research articles yet to be published.

Literature on behaviour change, communication, health communication, and social marketing was reviewed in parallel to the quantitative and qualitative data collection and guided by emergent themes from weekly discussions of the team. Messages deployed by the Ministry of Health were also reviewed to ensure consistency and alignment with the official information provided. In an iterative process of collecting primary data, reflecting as a team, and reviewing the literature, we developed new categories to include in the interview codebook. These included possible key themes to design the messages; values and key characteristics of the participants; possible preference of participants for the type of message [19,52]; and cultural traits identified in the interviews (i.e. individualistic, collectivistic, both) [53,54]. The coding process was iterative; we listened to the audio recordings several times at different moments and refined the coding, especially to identify subtle trends.

### Phase 2: drafting messages

This phase consisted of identifying message categories and developing the messages that would be evaluated in the third phase of the co-design process. Three local research team members participated in this second phase. The research team members independently listened to the interview audio recordings, read their transcripts, reviewed the data coded, and had meetings to identify emerging themes, in the light of the literature review findings and considering the interviewees’ message suggestions provided. Thanks to this process, the content of the mHealth vaccine intervention arms was decided. Afterwards, a fourth team member selected excerpts from the interview transcripts and made small language edits (in Spanish), to use some of the participants’ experiences in the design of the messages. Throughout this process, the lead author developed the first set of messages. The developed messages were further refined through an iterative process with the participation of the initial three team members. A preselection of messages to evaluate collectively was made.

### Phase 3: evaluation of the content of the messages

The content of the messages preselected in the second phase was evaluated through an online questionnaire developed for this study and a co-design workshop. The behavioural economists from the funding agency and the research team participated in this phase. The questionnaire included quantitative and qualitative questions. Using a five-point Likert Scale, participants were asked to evaluate each pre-selected message according to six criteria for effective communication [20,21]. We asked them to rate how timely, accurate, clear, concise, credible, and relevant each message was. We established scoring ranges and set a minimum score per message to qualify for preselection. The preselected messages were subsequently analysed qualitatively based on the results of two open-ended questions asking participants about possible unintended consequences and suggestions to improve each message.

This phase also included a two-hour co-design workshop carried out in Spanish that was held in parallel with the completion of the questionnaire. The workshop aimed to engage participants in the process of evaluating and refining messages. The session consisted of four main parts: (1) a review of the main findings of the formative studies, (2) a description of the co-design methodology used in the project, (3) a presentation of the content evaluation criteria, and (4) a participatory process to jointly improve some messages considering the six evaluation criteria. Participants read and listened to some of the messages and discussed them, considering the evaluation criteria. They also provided insights based on their prior experiences developing health behaviour change interventions.

The feedback obtained during the co-design workshop and the evaluation questionnaire’s mixed quantitative and qualitative results informed the pre-selection of some messages. These pre-selected messages were further edited by the lead author based on feedback from two local and two non-local team members.

### Phase 4: evaluation of the voices for the messages

The last phase of the co-design process entailed evaluating whose voice should be recorded for the national mHealth intervention. First, we identified potential voices of men and women for the audio messages. We searched for neutral voices to facilitate understanding and affinity by people from any region of the country. Then we recorded a sample of the messages, and subsequently, evaluated the recorded messages qualitatively and quantitatively.

The qualitative evaluation was conducted through short mobile phone interviews by the same three team members who carried out the formative mobile phone interviews. They called participants they had interviewed before. A sample of participants of the formative mobile phone interviews, who had expressed interest in providing additional suggestions for the design of the messages, were also invited to participate in the evaluation of the voices. The evaluation was intentionally done to ensure an equal representation of men and women. Protocols were developed to guide the recording of the audio messages, and the evaluation processes.

Foreseeing potential participant fatigue, as they had been involved in both the surveys and interviews of the formative research, in the qualitative evaluation we divided the sample into two groups, each comprised of women and men. One group evaluated the women’s voices of one of the message categories and the other the men’s voices of the other category of messages. In other words, participants listened to two versions of the same message delivered by different voices of men or women. After listening to each message, participants were asked if they liked the voice of the messenger, what feelings prompted the message, and if the messenger was trustworthy. They were also requested to describe the voice, and if the voice would inspire them to get vaccinated. Next, the participants were questioned about which of the two voices they preferred and to provide the reasons for their preference. Finally, suggestions to improve the messages were solicited from participants.

In contrast to the qualitative evaluation, the quantitative evaluation delivered through IVR had closed response options. This evaluation used the same voices as the qualitative evaluation. Participants were also divided into two groups to assess the voices, and the message categories were randomised to the participants. Lastly, the four phases of the message co-design process were also informed by insights from the research team that emerged during weekly reflexive meetings.

## Results

### Phase 1: formative research

#### Formative studies’ participants

Two surveys were deployed at the national level in the year 2022. A total of 367 people (209 women, 156 men, 2 non-binary) participated in the first survey deployed on March 12^th^, and 451 people (244 women, 199 men, 8 non-binary) in the second survey implemented on April 23^rd^. The mobile phone interviews were conducted in parallel to the second survey. A total of 36 people (19 women, 17 men, 0 non-binary) from four regions (i.e. Andean, Caribbean, Orinoquia, Pacific) of the country participated in the interviews. Some participants lived in rural areas (2 women, 4 men) and one of them was a Health Coordinator of a Pacific region Indigenous community. Four of the participants were Venezuelan immigrants (1 woman, 3 men). More detailed results from the formative studies will be presented in research articles that will be published shortly.

#### Vaccination status and intention to get vaccinated

Our studies inquired about the vaccination status of the survey and mobile phone interview participants. At the time these studies were carried out, the first booster was available to the public. Results show that most survey participants (*n *= 178 first survey, *n *= 207 second survey) had been fully vaccinated but had not taken the booster dose, and that a total of 11 interview participants had taken the first booster. In contrast, 10% of the respondents of the first survey (*n *= 367), 12% of the second survey participants (*n = *451), and 22.2% of interview participants (*n *= 8) had not been vaccinated. The survey findings also revealed the main motivation behind not wanting to get vaccinated was the opinion that the vaccine was not safe enough. This was corroborated during the mobile phone interviews.

Both surveys were conducted at the national level; however, the second survey disaggregated data by region. Results from the second survey show regional differences in vaccination status. It was found that the percentage of unvaccinated people was higher in the Pacific region (18%). In contrast, most of the unvaccinated interview participants were from the Andean region (*n* = 4) and some from the Pacific region (*n* = 2). The existence of regional differences and their effects on vaccination are observed in the following comments from mobile phone interview participants:

“Well, the experience of my vaccination … has been very good, it has been successful because, well, you know that by getting the vaccines you avoid contracting COVID.” *(Interviewee 5, man, works in the construction sector, urban, Andes region – translated from Spanish to English)*

We are in a region [Caribbean], in which many myths and things are made up, so all those things are how people allow themselves to be led by what grandparents and the older people say. *(Interviewee 34, woman, works in services sector, urban, Caribbean region – translated from Spanish to English)*

We do not share the issue of vaccines. We manage our own medicine (…) and it has worked for us, well, up to now it has worked well for us… we as indigenous populations… we manage our own traditional medicine and I think that up to now it has worked very well for us. *(Interview 13, man, indigenous health coordinator, rural, Pacific region – translated from Spanish to English)*

The surveys explicitly asked about participants’ intention to get vaccinated against COVID-19. A small sample of participants from the first (*n* = 21) and the second (*n* = 38) round of surveys responded with not wanting to receive it. Similarly, during the mobile phone interviews, some participants shared interest in getting their first vaccine dose (*n* = 3) or in getting the next COVID-19 vaccine dose (*n* = 14).

**Figure 2.**
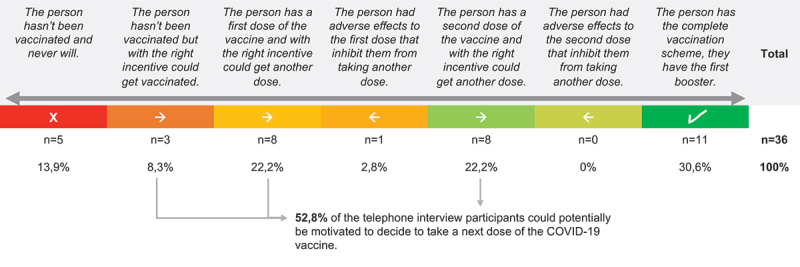
COVID-19 vaccination continuum of mobile phone interview participants.

We mapped participants’ intentions to vaccinate in a continuum from not being vaccinated and not having an interest in getting the COVID-19 vaccine, to being fully vaccinated and with the first booster (see Figure 2). This was done upon interpreting the mobile phone interview conversations. Developing this graph helped to identify the potential to motivate people to decide to take the next vaccine dose. For some, this would mean taking the first dose, for others, taking a booster dose.

#### Insights for the creation of the messages

Formative research results illustrated priority topics to inform message design. These topics included: 1) addressing the adverse effects of the vaccines, 2) clarifying that getting vaccinated reduced symptoms rather than curing the disease, and 3) providing more information about the effectiveness of the vaccines. Another important finding people expressed during the mobile phone interviews was that getting vaccinated against COVID-19 should be seen as a symbol of love and respect for family and friends, and as an act of social responsibility. Several participants also conveyed that vaccination should be an individual’s decision rather than a mandate. References to self-protection, faith, benefits of getting vaccinated, the vaccine development processes, transparency about the ingredients of the vaccines and about who benefited from the vaccination processes, as well as the need to get vaccinated to be able to return to open spaces (e.g. work, study) were also mentioned by the interview participants.

Overall, people expressed the need to receive more information. They also wanted to learn about the positive experiences of other people with the COVID-19 vaccination and recommended using emotional appeals and highlighting the individual and collective benefits of vaccination. Interview participants also provided recommendations to develop the messages. Regarding the need to transmit honesty, not only with veracity, a participant stated: ‘Speak to us with truth to know if the vaccines work or don’t work’ (Interviewee 1, woman, nurse and chef, urban, Caribbean region). Another participant (interviewee 32, woman, entrepreneur, urban, Caribbean region) mentioned that many people who used to not believe in vaccines changed their opinion once they saw a reduction in mortality. She stated that it was necessary to ‘ver para creer’ (translation: ‘to see to believe’), which is a common expression used in Colombia. Others mentioned that using anecdotes and experiences as narratives could be valuable to people in the country, for example, a participant expressed: ‘ … tell [us] positive experiences, perhaps they transmit tranquility, credibility … ’ (Interviewee 31, woman, accountant, urban, Pacific region).

In sum, the formative research results indicated that there was potential to incentivise people to get the next dose of the vaccine, emphasising the booster, but that challenges existed to motivate those unwilling participants to get vaccinated. To address these concerns, interview participants underlined the importance of receiving more information, but also of knowing about the experience of other people with the vaccine. During the internal research team discussions, the need to develop messages that could appeal to the general population while at the same time being sensitive to participants’ intersecting identities and diversity was also constantly highlighted. These findings were analysed in the light of evidence from the literature that showed that the use of messages based on facts [19], on the personal experiences of people [9,55], and facts combined with personal experiences [23], while considering individualistic and collectivist appeals [53,54], have been effectively used to generate changes in behavioural determinants, behaviour, and health outcomes.

### Phase 2: drafting messages

Based on the formative research findings and the weekly research reflexive discussions, the research team proposed to develop three categories of messages. Each category corresponded to an arm of the mHealth intervention for national deployment: (1) factual messages, (2) narrative messages, and (3) mixed messages. An additional fourth arm was proposed for the intervention, in this control arm, no message would be delivered (see Table 1). The factual messages would call for rationality by presenting information based on data provided by relevant and trusted sources. The narrative messages would appeal to emotions drawing on the COVID-19 vaccination experiences of participants of the mobile phone interviews, guaranteeing their anonymity. Finally, the mixed messages would combine messages based on data (factual) with messages based on experiences (narrative). Each message category would have several topics addressing barriers and facilitators to COVID-19 vaccination (see Table 1).

**Table 1.** Co-designed message categories and topics per intervention arm.

	Arm 1: Factual	Arm 2: Narrative	Arm 3: Mixed		Arm 4: Control
			Factual	Narrative	
1	Vaccination and infection	Vaccination and infection	Vaccination and infection	Vaccination and infection	No message
2	Vaccine effectiveness	Vaccine effectiveness and vaccination/infection	Vaccine effectiveness	Vaccine effectiveness and vaccination/infection	No message
3	Vaccine development process	Vaccine development process	Vaccine development process	Vaccine development process	No message
4	Vaccine ingredients	Protection of society/social responsibility	Vaccine ingredients	Protection of society/social responsibility	No message
5	Security and adverse effects	Security and adverse effects	Security and adverse effects	Security and adverse effects	No message
6	Booster dose	Freedom to choose and vaccine security	Booster dose	Freedom to choose and vaccine security	No message
7	Family protection and care	Family protection and care	Family protection and care	Family protection and care	No message

During this phase of the message co-design process, collective decisions were made regarding the structure of the messages, the voices that would be used to record the messages to evaluate in the next two phases and to determine the frequency of delivery of the messages. It was decided that the factual messages would be recorded by one person and would consist of a phrase, whereas the narrative messages would have the voices of a woman and a man. It was also decided that the structure of the narrative messages would include a standard brief introduction inspired by the common Colombian saying, ‘to see in order to believe’ but rephrased to say ‘hear in order to believe’, a narrated experience, and a brief closure phrase with a call to action, all in Spanish. For the factual messages, it was preferred to include a neutral voice that transmitted security and legitimacy. The factual messages also ended with a call to action to motivate people to get vaccinated. The narrative messages, in contrast, required a voice that transmitted emotions, closeness, and authenticity. It was suggested that using voices, with neutral accents that resonated with the voices of laypeople would be ideal. Regarding frequency, the team decided to transmit each factual and narrative message twice per week. For the mixed message, each factual and narrative message was transmitted once weekly. The intervention would last seven weeks. Details of the intervention will be reported in a forthcoming original research article.

Once the message categories and their topics were decided, 41 preliminary messages were created. To draft the factual messages, three team members searched for scientific evidence [56–58], COVID-19 reports by the Colombian Ministry of Health [59], and recommendations made by the United States Centers for Disease Control and Prevention (CDC) [60]. Other team members validated the data with scientifically trusted sources. In contrast, the narrative messages were created by taking excerpts from the transcribed mobile phone interviews. The narrated experience of these messages was to be recorded using the voice of a man or a woman depending on the gender of the interview participant who inspired it. For example, if the excerpt was taken from an interview with a man, the narrated experience of the message would be recorded by a man. The preliminary messages drafted by the lead author were then critically reviewed and edited by two other team members. The resulting messages were preselected to be evaluated in the next phase of the co-design process.

### Phase 3: evaluation of the content of the messages

An online questionnaire and co-design workshop were used to evaluate the content of the 29 pre-selected messages (9 factual, 20 narrative). The online questionnaire was completed by a total of 13 people (7 men, 6 women). Two of these people were from the IADB (1 man, 1 woman), three from the nonlocal partner university (3 men), seven (2 men, 5 women) from the local partner university, and one Latina intern from a nonlocal university doing her master studies practicum with the project. These evaluation participants were experts in public health, communication, social marketing, behavioural economics, bioethics, epidemiology, sociology, literature, and psychology. The evaluation data analysis was carried out by two local team members.

The questionnaire evaluation focused on six evaluation criteria (timeliness, accuracy, clarity, conciseness, credibility, and relevance). Based on the number of participants and considering the minimum (1-point Likert Scale x 6 criteria x 13 people = 78), medium (3-point Likert Scale x 6 criteria x 13 people = 234), and maximum (5-point Likert Scale x 6 criteria x 13 people = 390) scores a message could have across all six evaluation criteria, it was determined that a message should have a total score of at least 234 to be qualified for selection. None of the messages had a score lower than the minimum established. The lowest score obtained was 294 for a narrative message focused on the adverse effects of the vaccine. When a message category had two or more options of messages, the message that had the higher rate was prioritised for selection consideration. The messages with higher scores per theme were selected. For the themes where only one message option was evaluated, the message was improved based on the qualitative feedback provided by the content evaluation questionnaire participants, as will be described next.

The evaluation questionnaire included some open-response questions. All the participants provided critical qualitative feedback to improve the messages. Examples of comments received included shortening the messages, using phrases such as ‘3 out of 4 people …’ instead of percentages, including a call to action at the end of the messages, and minimising the use of technical jargon. Having a message focused exclusively on the booster dose and using positive framing by underlining the benefits of the vaccines was also recommended.

A suggestion received for the narrative messages referring to culture is highlighted. In Colombia, where most of the population identifies as Catholic or Christian [61], it is common to find people referring to God in daily expressions. One of the evaluated messages used several references to God, as expressed by the interview participant (see below). However, one of the evaluation participants expressed that mentioning the words ‘God’ and ‘Jesus’ too many times could generate adverse reactions in people with different beliefs. The message was edited as suggested; however, it is important to note the person who made this suggestion was nonlocal.

I thought that my children were going to physically deteriorate. I said my God, Lord, in the name of Jesus, let nothing happen to them. And well, I took all five of them [my children] and gave them the vaccine. And when I arrived [at the house], everyone was jumping, when I had thought that everyone was going to fall into bed with a fever, with discomfort… They didn’t have anything [any adverse effect], thank God, they only felt sleepy. The vaccine made them sleepy; it sent them to bed to sleep. And after a while, they were already standing up bothering my life and that’s it [she said laughing].” (Interviewee 35, woman, mother, unemployed, urban, Caribbean region)

Lastly, some participants recommended avoiding the overemphasis on people getting vaccinated just because others have, as it could make some people believe that there’s already an appropriate level of population protection or herd immunity that might discourage vaccination. Additionally, various mobile phone interview participants recommended sharing examples of other vaccines (e.g. rubeola, measles, meningococcus) to motivate COVID-19 vaccination; however, expert feedback received during the content evaluation phase suggested otherwise. As a result, other vaccination examples were removed from the messages, which, in turn, helped shorten them in length.

The co-design workshop also provided important insights into editing the messages. A total of 12 people (5 men, 7 women) participated in this two-hour workshop held on 14 June 2022. Participants included behavioural economists from the funding agency’s office in the United States (*n* = 3) and in Colombia (*n* = 2), as well as research team members (*n* = 7). Suggestions made during the workshop included avoiding using examples of other vaccines, restraining from using negative phrases; shortening the messages; using general data for the factual messages instead of presenting data focused on specific age ranges; and avoiding reinforcing myths or content related to misinformation, even in a negative framing (e.g. chips as ingredients of the vaccines). Making subtle changes to the order of the content of the factual messages, and specific words, was also recommended. However, the content of the messages was overall deemed appropriate.

The workshop participants considered the voices of the text messages as appropriate to use for the mHealth intervention. Two participants mentioned that the recording of the factual messages appeared to be made by a journalist and highlighted that not many people trusted the news, which could be a limitation to engaging some study participants. Using a voice like that of the factual messages for the introduction and closing phrases of the narrative messages was suggested, as well as recording the audio messages with more intonation.

Based on the results of the evaluation questionnaire and feedback provided during the co-design workshop, some messages were selected and further refined by the team. Final approval was made by the local and nonlocal co-investigators of the project.

### Phase 4: evaluation of the voices for the messages

We conducted qualitative and quantitative evaluations of the voices of the factual and narrative messages. Prior to the evaluation, the local research team searched for potential voices among acquaintances and selected those that transmitted positive emotions (e.g. trust, warmth) and authenticity. The factual messages’ evaluation consisted of comparing the voice of an expert medical doctor and epidemiologist (Voice A) with that of a journalist (Voice B). For the narrative messages, the voices of two young men, one that resembles the voice of national television actors (Voice A) and another of a young man with a neutral voice (Voice B) were assessed.

**Figure 3.**
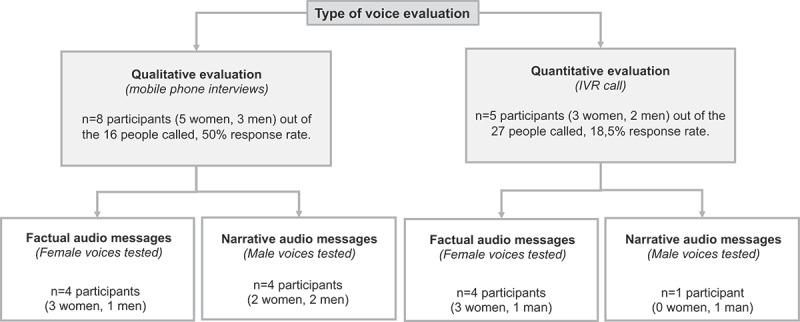
Qualitative and quantitative voice evaluation participants.

The qualitative evaluation of the voices was conducted by eight of the 16 individuals (5 women, 3 men) that were contacted. The factual message voices were assessed by four people (3 women, 1 man), whereas the narrative message voices were evaluated by four people (2 women, 2 men). The mobile phone interviews lasted an average of 11 minutes. The quantitative voice evaluation was completed by 5 of the 27 individuals (3 women, 2 men) contacted. A total of 4 people (3 women, 1 man) evaluated the voices for factual messages and one person (a man) the voice for the narrative message. The evaluation through IVR lasted an average of 7 minutes. See a summary of the participants per evaluation in Figure 3.

The results of the qualitative and quantitative evaluation of the voices for the factual messages showed a preference for Voice B. Two women preferred Voice A because it was calm and trustworthy. However, one of these participants said that although she preferred Voice A, she considered that Voice B would be more effective because it transmitted alarm and fear, and sometimes people responded better to that. The two other participants preferred Voice B. The man selected it because he perceived it as more concrete and direct. A woman stated that she had no interest in getting vaccinated and that she normally did not trust anyone’s voice. Although she didn’t trust any of the options of voices presented, she believed Voice B could be more effective. She stated: *‘Sometimes people believe journalists more. Sometimes you go to the [medical] doctor, and they say one thing, and then they say another, then they contradict themselves’* (Qualitative evaluation, factual voice, interview 3, woman). Similarly, the man who expressed not having the intention to get vaccinated indicated that people wouldn’t react well if an expert delivered the messages. He said that people *‘don’t pay attention to a [medical] doctor, they pay more attention to a third person… [they believe] that [medical] doctors kill, so being a [medical] doctor, they are not going to pay attention to her’*. (Qualitative evaluation, factual voice, interview 2, man). Based on this evaluation, Voice B was selected.

The evaluation of the voices for the narrative messages resulted in the selection of Voice B (layperson). No clear consensus was reached on the qualitative evaluation; however, the quantitative evaluation showed a preference for Voice B, therefore its selection. Gender differences in voice preference were observed in the qualitative evaluation of the narrative messages. Men preferred Voice B because it sounded clearer, more serious, and transmitted more trust, whereas women preferred Voice A because, in their opinion, it was more positive, manly, stronger, and transmitted more trust. The evaluation using IVR helped to decide the final voice selection and to improve the quality of the audio.

The last highlighted aspect of the co-design process is the research team’s weekly meetings, which were permanent and done in parallel with all the research phases. Having a multidisciplinary team of experts was valuable to the co-design process as the concepts, insights, and critical ideas that emerged from the discussions helped to improve the research processes and the content of the messages. In these meetings, for instance, the research team discussed preliminary findings considering social, cultural, and economic aspects and the effects of the evolving vaccination context on people’s intention to vaccinate. These discussions also revolved around how the gendered experiences and identities of the researchers influenced the data collection and interpretation processes, as well as the design of the messages. The need to decentralise the research and message design processes from the view of people living in the capital city and to elevate the voices and experiences of people from more peripheral regions or who represented minority groups (e.g. Venezuelan migrants and indigenous populations) was constantly discussed by the team.

### Co-designed messages

The co-design process described in this article led to the production of a total of 14 evidence-based COVID-19 vaccination audio messages. The scripts of these messages are presented in Table 2. The messages were recorded various times to ensure that the tone reinforced the content and that positive feelings were transmitted in both the factual and narrative messages. For example, when recording the narrative messages, special attention was paid to emulating as closely as possible the real-life experience that inspired the message (e.g. love for the family). The average length of the factual messages was 68 seconds and of the narrative messages of 48,4 seconds. These messages will be used in an mHealth intervention to be implemented at the national level in Colombia. The results of the implemented intervention will be described in a different research article.

**Table 2.** Factual and narrative co-designed messages.

No.	Structure of the message	Message categories and topics in English (translated)	Message categories and topics in Spanish (original)	Sender
***Factual Messages***				
1	Vaccination and infection			
	Whole message	Did you know that getting vaccinated against COVID-19 reduces the risk of developing severe symptoms, needing hospitalization, and dying?	¿Sabía usted que vacunarse contra COVID-19 reduce el riesgo de desarrollar síntomas graves, de necesitar hospitalización y de morir?	Woman journalist’s voice (Voice B)
		Vaccines against COVID-19, as for other diseases, help so that if you do get sick, the symptoms are milder than if you did not have the vaccine.	Las vacunas contra COVID-19, al igual que para otras enfermedades, ayudan a que si te enfermas, los síntomas sean más suaves que si no tuvieras la vacuna.	
		For example, according to data from the Colombian Ministry of Health, people who have been vaccinated against COVID-19 have three times less risk of hospitalization than unvaccinated people.	Por ejemplo, de acuerdo a datos del Ministerio de Salud de Colombia, las personas que han sido vacunadas contra COVID-19, tienen tres veces menos riesgo de hospitalización que las personas no vacunadas.	
		If you have not yet been vaccinated or are missing a	Si aún no se ha vacunado o le falta alguna dosis, recuerde hacerlo lo	
		dose, remember to do so as soon as possible.	antes posible.	
2	Vaccine effectiveness			
	Whole message	Did you know that as of June 2022, eight out of ten Colombians had received at least one dose of the COVID-19 vaccine and that, thanks to this, more than 36,000 lives have been saved?	¿Sabía usted que a junio de 2022, 8 de cada 10 colombianos se había aplicado al menos una dosis de la vacuna contra COVID-19 y que, gracias a esta, se han salvado más de 36,000 vidas?	Woman journalist’s voice (Voice B)
		In addition, more than half of the people vaccinated against COVID-19 did not need hospitalization when they became infected.	Además, más de la mitad de las personas vacunadas contra COVID-19 no necesitaron hospitalización cuando se contagiaron.	
		If you have not yet been vaccinated, remember to do it soon because we are at a new peak of COVID-19. If you just got COVID-19, remember to wait 30 days to get vaccinated.	Si aún no se ha vacunado, recuerde hacerlo pronto porque estamos en un nuevo pico de COVI-19. Si le acaba de dar COVID-19, recuerde esperar 30 días para vacunarse.	
		It is necessary to get vaccinated because the defenses that your body generated during that infection will not be enough to fight a new attack of the virus.	Es necesario vacunarse porque las defensas que generó su cuerpo durante esa infección, no serán suficientes para combatir un nuevo ataque del virus.	
3	Vaccine development process			
	Whole message	Did you know that COVID-19 vaccines were developed following rigorous approval and safety monitoring processes around the world?	¿Sabía usted que las vacunas contra COVID-19 se desarrollaron siguiendo rigurosos procesos de aprobación y monitoreo de su seguridad en todo el mundo?	Woman journalist’s voice (Voice B)
		It was faster to create the vaccine against COVID-19 than for other diseases because we have more technology, because there were already scientific studies to design vaccines against other similar diseases, and because this time the scientific community around the world worked collaboratively. Thanks to all this, as of June 2022, more than 42 million people in Colombia have been vaccinated with at least one dose of the vaccine against COVID-19.	Fue más rápido crear la vacuna contra COVID-19 que para otras enfermedades porque tenemos más tecnología, porque ya había estudios científicos para diseñar vacunas contra otras enfermedades parecidas y, porque esta vez la comunidad científica de todo el mundo trabajó colaborativamente. Gracias a todo esto, a junio de 2022 más de 42 millones de personas en Colombia han sido vacunadas con al menos una dosis de la vacuna contra COVID-19.	
		Vaccinating ourselves protects us all. If you have not yet been vaccinated or are missing a dose, remember to do so as soon as possible.	Vacunándonos nos protegemos todos. Si aún no se ha vacunado o le falta alguna dosis, recuerde hacerlo lo antes posible.	
4	Vaccine ingredients			
	Whole message	Have you ever wondered what COVID-19 vaccines are made of?Regardless of the brand, because they all work and are safe, these vaccines do not contain eggs, gluten, or preservatives. They also do not have metals, plastics, and electronic equipment. Vaccines teach the body to build defenses against future attacks by the virus.If you have not yet been vaccinated against COVID-19 or are missing a dose, remember to do so as soon as possible.	¿Alguna vez se ha preguntado de qué están hechas las vacunas contra COVID-19?Sin importar la marca, porque todas sirven y son seguras, éstas vacunas no contienen huevos, gluten, ni preservativos. Tampoco tienen metales, plásticos, ni equipos electrónicos. Las vacunas le enseñan al cuerpo a generar defensas contra futuros ataques del virus.Si aún no se ha vacunado contra COVID-19 o le falta alguna dosis, recuerde hacerlo lo antes posible.	Woman journalist’s voice (Voice B)
5	Security and adverse effects			
	Whole message	Did you know that COVID-19 vaccines have prevented three out of five people infected with COVID-19 from being hospitalized?	¿Sabía usted que las vacunas contra COVID-19 han evitado que tres de cada cinco personas infectadas con COVID-19 sean hospitalizadas?	Woman journalist’s voice (Voice B)
		But, getting vaccinated, like any change that is made in the body, can have an effect.	Pero, vacunarse, como cualquier cambio que se hace en el cuerpo puede tener un efecto.	
		In studies conducted to ensure that the vaccines were safe, involving thousands of people, it was found that some people reported temporary effects of the vaccines, including temporary pain in the arm where the vaccine was given, some tiredness, fever or nausea, and in some women, alterations in menstruation.Remember that the possible discomfort caused by the vaccine is temporary and that the benefits of the vaccine are even greater. It could save your life.If you have not yet been vaccinated or are missing a dose, remember to do so as soon as possible.	En los estudios que se hicieron para garantizar que las vacunas fueran seguras, donde participaron miles de personas, se encontró que algunas personas reportaron efectos temporales de las vacunas entre estos, dolor transitorio en el brazo en el que se aplicó la vacuna, un poco de cansancio, fiebre o náuseas y en algunas mujeres, alteraciones en la menstruación.Recuerde que los posibles malestares causados por la vacuna son temporales y que los beneficios de la vacuna son aún mayores. Podría salvarle la vida.Si aún no se ha vacunado o le falta alguna dosis, recuerde hacerlo lo antes posible.	
6	Booster dose			
	Whole message	Did you know that COVID-19 booster vaccines help maintain protection against severe symptoms of the disease?	¿Sabía usted que las vacunas de refuerzo contra COVID-19, ayudan a mantener la protección contra los síntomas graves de la enfermedad?	
		When we get booster doses, we increase our body’s immunity and its ability to fight a new attack from the virus. This is important because, as with other vaccines, the protection of vaccines against COVID-19 decreases as time passes and the disease changes.Remember that the first booster dose is available to everyone over 18 years of age and is given four months after completing the vaccination schedule. If you are missing the booster dose, remember to get it as soon as possible.	Cuando nos aplicamos las dosis de refuerzo, incrementamos la inmunidad de nuestro cuerpo y su capacidad para combatir un nuevo ataque del virus. Esto es importante porque, así como sucede con otras vacunas, la protección de las vacunas contra COVID-19 va disminuyendo en la medida que pasa el tiempo y la enfermedad va cambiando.Recuerde que la primera dosis de refuerzo está disponible para todas las personas mayores de 18 años y, se aplica cuatro meses después de haber completado el esquema de vacunación.Si le falta la dosis de refuerzo, recuerde hacerlo lo antes posible.	Woman journalist’s voice (Voice B)
7	Family protection and care			
	Whole message	Did you know that studies conducted in Colombia and other countries have found that vaccines against COVID-19 help to protect adults, adolescents, boys, and girls with whom we live?	¿Sabía usted que estudios realizados en Colombia y otros países han encontrado que las vacunas contra COVID-19 ayudan a proteger a los adultos, adolescentes, niños y niñas con quienes convivimos?	Woman journalist’s voice (Voice B)
		Thanks to these vaccines, more than 36,000 lives were saved last year in Colombia.	Gracias a estas vacunas, el año pasado se salvaron más de 36,000 vidas en Colombia.	
		Vaccinating ourselves protects us all. If you have not yet been vaccinated or are missing a dose, remember to do so as soon as possible.	Vacunándonos nos protegemos todos. Si aún no se ha vacunado o le falta alguna dosis, recuerde hacerlo lo antes posible.	
***Narrative Messages***				
8	Vaccination and infection			
	Start and contextualization	Hearing is Believing. We interviewed people from all over Colombia, and do you know what they told us about vaccination against COVID-19?	Oír para creer, entrevistamos a personas de todo Colombia y, ¿saben qué nos contaron sobre la vacunación contra COVID-19?	Young man’ voice (Voice B)
	Core message	“At the beginning of the pandemic, it was a bit difficult. One was used to a work routine and social life, and, well, suddenly, for health reasons, everything changed. But precisely, the vaccine is to ensure that, if you do get infected, it won’t hit you so hard.”	“Al principio de la pandemia fue un poco difícil, uno venía acostumbrado a una rutina de trabajo, vida social y, pues, de repente, por cuestiones de salud, todo cambió. Pero, precisamente, la vacuna es para que, si llegado el caso te llegas a contagiar, no te pegue tan fuerte”.	Woman’s voice (Not tested)
	Ending with a call to action	Vaccines reduce the risk of developing severe symptoms. If you have not yet been vaccinated or are missing a dose, remember to visit the nearest vaccination center.	Las vacunas reducen el riesgo de desarrollar síntomas graves. Si aún no te has vacunado o te falta alguna dosis, recuerda visitar el punto de vacunación más cercano.	Young man’s voice (Voice B)
9	Vaccine effectiveness and vaccination/infection			
	Start and contextualization	Hearing is Believing. We interviewed people from all over Colombia, and do you know what they told us about vaccination against COVID-19?	Oír para creer, entrevistamos a personas de todo Colombia y, ¿saben qué nos contaron sobre la vacunación contra COVID-19?	Woman journalist’s voice
	Core message	“I got COVID twice and it left me with sequelae: with many respiratory problems and now I am in treatment. So, in order to get vaccinated I had to ask my doctors and they told me that there was no problem, so I got vaccinated with two doses and I am waiting for the third one. And I tell you that, to my surprise, I thought that due to my sequelae, I was going to have some reaction from the vaccine, and hey, no! I didn’t get me anything.”	“A mí me dio dos veces COVID y me dejó secuelas: con muchos problemas respiratorios y ahora estoy en tratamiento. Entonces, para poder vacunarme tuve que preguntarles a mis médicos y ellos me dijeron que no había ningún problema, así que me vacuné con dos dosis y estoy pendiente de la tercera. Y le cuento que, para mi sorpresa, pensé que por mis secuelas iba a tener alguna reacción por la vacuna y ¡oiga, no! no me dio nada.”	Older man’ voice(Not tested)
	Ending with a call to action	Even if you have been infected with COVID-19 you can also get vaccinated. If you have not yet been vaccinated or are missing a dose, remember to visit the nearest vaccination center.	Así te hayas enfermado de COVID-19 también puedes vacunarte. Si aún no te has vacunado o te falta alguna dosis, recuerda visitar el punto de vacunación más cercano.	Woman journalist’s voice
10	Vaccine development process			
	Start and contextualization	Hearing is Believing. We interviewed people from all over Colombia, and do you know what they told us about vaccination against COVID-19?	Oír para creer, entrevistamos a personas de todo Colombia y, ¿saben qué nos contaron sobre la vacunación contra COVID-19?	Young man’s voice (Voice B)
	Core message	“Vaccination, for me, is something very important. It really is that with the crisis that occurred, it was very impressive that they released the vaccines so quickly! When I found out about the vaccines, I felt happy, because, fortunately, despite working in a hospital, I did not get infected. With the vaccine, I became more comfortable at work. I am a surgical instrument assistant, and I have always believed in science because it isavailable to us, to act quickly in these situations”.	“La vacunación, para mí, es algo muy importante. Realmente es que con la crisis que hubo, ¡fue muy impresionante que hayan sacado tan rápido las vacunas! Cuando me enteré de las vacunas, me sentí feliz, porque, afortunadamente, a pesar de estar trabajando en un hospital, no me contagié. Ya con la vacuna estaba más tranquila en mi trabajo. Soy instrumentadora quirúrgica, y siempre he creído en la ciencia, porque ella está disponible para nosotros, para actuar rápido ante estas situaciones.”	Woman’s voice(Not tested)
	Ending with a call to action	Vaccines work. If you have not yet been vaccinated or are missing a dose, remember to visit the nearest vaccination center.	Las vacunas funcionan. Si aún no te has vacunado o te falta alguna dosis, recuerda visitar el punto de vacunación más cercano.	Young man’s voice (Voice B)
11	Protection of society/social responsibility			
	Start and contextualization	Hearing is Believing. We interviewed people from all over Colombia, and do you know what they told us about vaccination against COVID-19?	Oír para creer, entrevistamos a personas de todo Colombia y, ¿saben qué nos contaron sobre la vacunación contra COVID-19?	Young man’s voice (Voice B)
	Core message	“My decision was always to get vaccinated for prevention, health, respect to my family and at a societal level because I believe that it is a matter of social responsibility that everyone gets vaccinated; I believe that we all have a risk, we do not know how the virus will react in each person and well, I believe that we should be motivated by the love to our family and to ourselves, and that, in my case, I have to be healthy to protect them in some way; so I already have the three doses of the vaccine and all is going well, I didn’t have any type of reaction in any of the three doses. My family is also vaccinated and none of them had adverse effects”.	“Mi decisión siempre fue vacunarme por prevención, por salud, por respeto a mi familia y a nivel social, porque creo que es algo de responsabilidad social, que todos debemos estar vacunados; considero que todos tenemos un riesgo, no sabemos cómo va a reaccionar el virus en cada persona y bueno, creo que debería motivarnos, principalmente, el amor a nuestra familia y a nosotros mismos, y por eso, en mi caso, debo estar bien y protegerlos de cierta manera; entonces, ya tengo las tres dosis de la vacuna y súper bien, no tuve ningún tipo de reacción en ninguna de las tres dosis. También mi familia está vacunada y ninguno tuvo síntomas”.	Young man’s voice (Voice B)
	Ending with a call to action	Taking care of ourselves is everyone’s responsibility. If you have not yet been vaccinated or you are missing a dose, remember to visit the nearest vaccination center.	Cuidarnos es responsabilidad de todos. Si aún no te has vacunado o te falta alguna dosis, recuerda visitar el punto de vacunación más cercano.	Young man’s voice (Voice B)
12	Security and adverse effects			
	Start and contextualization	Hearing is Believing. We interviewed people from all over Colombia, and do you know what they told us about vaccination against COVID-19?	Oír para creer, entrevistamos a personas de todo Colombia y, ¿saben qué nos contaron sobre la vacunación contra COVID-19?	Young man’s voice (Voice B)
	Core message	“Right now, I am unemployed because due to the pandemic, the school where I worked had to close. The pandemic changed our lives 100%… and vaccination was a way to survive this virus because we knew that if it hit us, we could survive with the vaccine, so, for everyone’s wellbeing, in my family we are all vaccinated. In my case, I have all the doses and the booster. I was afraid to get this last one because of all the comments I heard about that vaccine, but no, it was normal. It went super well for my husband and me who got it.”	“En este momento estoy desempleada porque debido a la pandemia el colegio donde yo trabajaba tuvo que cerrar. La pandemia nos cambió la vida 100%… y la vacunación fue una manera de sobrevivir a este virus, porque sabíamos que, si nos daba, podríamos sobrevivir con la vacuna, entonces, por el bienestar de todos, en mi familia todos estamos vacunados. En mi caso, tengo todas las dosis y la de refuerzo. Tuve miedo de ponerme esta última por todos los comentarios que escuchaba de esa vacuna, pero no, normal. Nos fue super bien a mi esposo y a mí que nos la pusimos.”	Woman’s voice(Not tested)
	Ending with a call to action	Vaccines are safe. If you have not yet been vaccinated or are missing a dose, remember to visit the nearest vaccination center.	Las vacunas son seguras. Si aún no te has vacunado o te falta alguna dosis, recuerda visitar el punto de vacunación más cercano.	Young man’s voice (Voice B)
13	Freedom to choose and vaccine security			
	Start and contextualization	Hearing is Believing. We interviewed people from all over Colombia, and do you know what they told us about vaccination against COVID-19?	Oír para creer, entrevistamos a personas de todo Colombia y, ¿saben qué nos contaron sobre la vacunación contra COVID-19?	Woman journalist’s voice
	Core message	“The vaccine has worked well, after the vaccination, the number of deaths due to COVID-19 decreased. So, I believe that people should get vaccinated, well, to be safer, to live more peacefully, and continue living everyday life; and for that, we must do our part.”	“La vacuna ha servido mucho, después de la vacunación, disminuyó el número de muertes por COVID-19. Entonces, creo yo que la gente debería vacunarse, pues, para estar más seguros, para vivir más tranquilos y seguir viviendo la cotidianidad; y para eso tenemos que poner de nuestra parte.”	Young man’s voice (Voice B)
	Ending with a call to action	By vaccinating against COVID-19 we are safer. If you have not yet been vaccinated or are missing a dose, remember to visit the nearest vaccination center.	Vacunándonos contra COVID-19 estamos más seguros. Si aún no te has vacunado o te falta alguna dosis, recuerda visitar el punto de vacunación más cercano.	Woman journalist’s voice
14	Family protection and care			
	Start and contextualization	Hearing is Believing. We interviewed people from all over Colombia, and do you know what they told us about vaccination against COVID-19?	Oír para creer, entrevistamos a personas de todo Colombia y, ¿saben qué nos contaron sobre la vacunación contra COVID-19?	Young man’s voice (Voice B)
	Core message	“My five-year-old son is already vaccinated too, well, I didn’t want him to get sick either, and since he was going to school and going out with his friends, that’s why. I love my children so much! I adore them and would give my life for them, and that’s why I got vaccinated, because maybe one day I go out, get infected, I come and hug them or kiss them and spread the virus to them… I had to do it for them.”	“Mi hijo de cinco añitos ya está vacunado también, pues, porque tampoco quería que él se enfermara y como estaba yendo al colegio y salía con amiguitos, entonces, por eso. ¡Yo quiero tanto a mis hijos! Los adoro y daría la vida por ellos, y por eso me vacuné, porque que tal un día salga, me contagie, yo llegue y los abrace o los bese y les pase el virus … yo tenía que hacerlo por ellos.”	Woman’s voice(Not tested)
	Ending with a call to action	Protect yourself and your family. If you have not yet been vaccinated or are missing a dose, remember to visit the nearest vaccination center.	Protégete y protege a tu familia. Si aún no te has vacunado o te falta alguna dosis, recuerda visitar el punto de vacunación más cercano.	Young man’s voice (Voice B)

## Discussion

This article describes the process used to co-design messages to promote voluntary uptake of COVID-19 vaccines in an adult population in Colombia. The process included conducting quantitative and qualitative formative studies, drafting messages, and evaluating the content and possible voices to use for the audio messages. The developed messages are to be deployed in a cohort of individuals through IVR mobile phone surveys.

The results of the message co-design process show that using messages that appeal to rationality and emotions and that combine individualistic and collectivistic values is important. Based on our research, addressing the vaccine development process, the vaccines’ effectiveness, risks, and adverse effects, as well as people’s possibility to decide on vaccination, and the effects of vaccination on family and society’s protection were deemed important. This is similar to findings from a study about COVID-19 vaccination in Colombia, Ecuador, and Venezuela which found that vaccine safety, and side and long-term effects were major concerns of people across the three countries [10]. Based on these results, the scholars recommend using communication to address COVID-19 vaccine hesitancy [10]. While information-based messages are necessary [20,21], using narrative messages that convey positive experiences and emotions can help create closer links between people and health [62], rather than using messages appealing to fear which the evidence shows do not always encourage people to get vaccinated [63]. The effects of the COVID-19 pandemic generated high emotional and mental health distress [64] which made using vaccination messages appealing to negative emotions (e.g. fear) unethical and inappropriate. This is consistent with calls to incorporate higher ethical standards when addressing the COVID-19 pandemic [65,66]. Consequently, in co-designing the messages presented in this article, the emotional and mental health of people as the COVID-19 pandemic progressed were considered.

The iterative co-design process involving diverse stakeholders helped to develop messages with a higher potential to be effective in fostering COVID-19 vaccination. Changes in people’s COVID-19 vaccine hesitancy determinants and the public health landscape were identified through the continued engagement of the co-design process participants and the constant review of evidence generated by trusted sources. The timely identification of ongoing needs helped to refine the messages continuously and improve their quality. Refining the quality of the messages would not have happened if the messages had been designed at a specific time and without the use of participatory approaches. These types of approaches have proven to be successful in developing health interventions in LMICs [29,30]. Respect for participants’ voices and decision-making processes related to COVID-19 vaccines and vaccination was fundamental throughout the co-design process we carried out, especially in the qualitative components and message script drafting.

The experiences and opinions of the people who would receive the intervention were at the centre of this co-design process. While expert opinion guided the research processes, the research team was committed to avoiding top-down paternalistic approaches. The research methodology described in this article articulates well with global calls to develop appropriate responses to population needs through the use of participatory approaches [67,68]. The co-design of interventions requires more financial and non-financial resources (e.g. time, money, people) than following traditional research processes that value pre-designed solutions [67]; however, investing in participatory approaches involving relevant stakeholders could increase the possibility for an intervention to be accepted and successful in influencing health behaviours [67]. Given the limited resources available to address health needs, including during emergencies such as the COVID-19 pandemic, using co-design approaches could help to make better use of public health resources.

Fostering sustainable behaviour change to address public health priorities requires investing in interventions that have the greatest potential for impact. Co-design methods offer opportunities to develop more effective interventions that result in positive health outcomes. The use of mobile phones in co-design processes could help to engage hard-to-reach populations at lower costs than using in-person methods. Nevertheless, using digital technologies also has disadvantages, such as the limited possibility of establishing a deeper connection with participants as technology tools might be seen as a barrier to having more genuine and meaningful communication. Similar to what other scholars have posited, more research exploring the cost-effectiveness and behaviour change effects of using co-design methods in message and intervention development is commended [69].

### Strengths and limitations of this study

A strength of the research process described in this article was the use of mixed methods to compare and validate the research findings, as well as to refine the messages as seen in the formative research and the message evaluation. Another strength of the co-design methodologies mentioned in this article was the way it allowed us to consider Colombia’s diversity (e.g. vaccination experiences, cultures, structures, language use differences), the possible needs of underserved populations (e.g. low socio-economic status, migrants), and the varied experiences of people based on their intersecting identities to develop messages that could be delivered to multiple audiences while at the same time being relevant to them. This contrasts with interventions that do not consider the identities and experiences of their audiences, and only focus on providing information.

A limitation of this study was that the quality of the mobile network in the country varies between rural and urban areas, with some remote areas having an unstable connection, making it more difficult to reach some populations. To address this and based on prior experiences of the research team, measures were taken to ensure a minimum participant sample per region of the country. Our efforts led to positive results, for instance, in the participation of an indigenous leader from a rural area of the Pacific region. Despite these measures, potential participants might have been missed. The COVID-19 vaccination messages developed through the process described in this article were limited in that they were not tailored to segmented audiences. Ideally, in the absence of time and budget restrictions, vaccination health messages should be tailored to reach different segments of the population prioritised [52], frequently adjusted to address changing people’s perceptions, knowledge, and experiences with vaccination and with the disease [70], and adapted during implementation according to infection wave changes [70]. However, the mobile phone interviews carried out to inform the development of the messages helped to identify key commonalities among participants with varied backgrounds, which were incorporated into the co-design messages meant to resonate with diverse audiences. Moreover, given the small sample of participants in the mobile phone interviews, findings from this study might not represent the perceptions, knowledge, and experiences of all people who would receive the co-developed messages through an mHealth intervention. Triangulating the results of the quantitative and qualitative formative studies, and the iterative co-design process informed by expert feedback and scientific and grey literature, enabled the research team to address potential gaps in the data collected in the formative research interviews. It is suggested that in future studies, more in-depth qualitative research is conducted to understand the specific characteristics of segmented audiences to develop more tailored messages, not only for adults but also for children and adolescents.

Another limitation of this study relates to the feedback provided in the content evaluation phase. The recommendations obtained through the online questionnaire showed that a few participants mentioned ideas that were shared by others during the co-design workshop. This limitation was identified in a timely manner by the research team, which enabled a balanced inclusion of the ideas provided by participants at all phases of the process. It is suggested that future studies, implementing a similar methodology, conduct individual assessments before any research process involving other participants to reduce potential response bias. Lastly, the voice evaluation did not test the woman’s voice in the narrative messages nor the older man’s voice that was used in one of these experience-based messages due to time and budgetary restrictions. These voices, however, were carefully examined and assessed by the research team and were deemed appropriate.

## Conclusion

This research article shows the possibilities and strengths of co-designing evidence-based health messages for adult populations using mobile phones in LMICs contexts and during health emergencies such as the COVID-19 pandemic. The methodologies and lessons learned from this work could inform future health emergency responses. Governments, funding agencies, and organisations involved in disease response are encouraged to use message co-design processes to develop health messages using mHealth technologies. Moving beyond top-down paternalistic perspectives that delimit the focus of health interventions and the content of health messages to embrace more context-relevant, participatory, people-centred, and reflexive multidisciplinary approaches could help develop solutions that are more responsive to the health needs of populations and public health priorities. Co-designing health messages requires constant commitment and allocation of resources to attain positive results. Based on the experiences described in this article, we recommend others invest in co-designing health messages that would be delivered via mHealth interventions or other channels as they have the potential to influence positive health behaviours and ultimately improve health outcomes.

## Paper context

The COVID-19 pandemic represents a global priority. Effective communication could help increase COVID-19 vaccine uptake. Using participatory methods could help in developing messages with greater potential for behaviour change impact. Limited evidence describing how vaccination promotion messages are developed exists. This article describes the methodology used to co-develop COVID-19 vaccination messages to be deployed to a cohort of adults in Colombia via interactive voice response (IVR) mobile phone surveys.
